# Overnight exposure to high altitude in pulmonary hypertension: adverse events and effect of oxygen therapy

**DOI:** 10.1093/eurheartj/ehad789

**Published:** 2023-12-11

**Authors:** Simon R Schneider, Julian Müller, Meret Bauer, Laura Mayer, Lea Lüönd, Tanja Ulrich, Michael Furian, Aglaia Forrer, Arcangelo Carta, Esther I Schwarz, Konrad E Bloch, Mona Lichtblau, Silvia Ulrich

**Affiliations:** Department of Pulmonology, University Hospital Zurich, Rämistrasse 100, CH-8091 Zürich, Switzerland; Department of Pulmonology, University Hospital Zurich, Rämistrasse 100, CH-8091 Zürich, Switzerland; Department of Pulmonology, University Hospital Zurich, Rämistrasse 100, CH-8091 Zürich, Switzerland; Department of Pulmonology, University Hospital Zurich, Rämistrasse 100, CH-8091 Zürich, Switzerland; Department of Pulmonology, University Hospital Zurich, Rämistrasse 100, CH-8091 Zürich, Switzerland; Department of Pulmonology, University Hospital Zurich, Rämistrasse 100, CH-8091 Zürich, Switzerland; Department of Pulmonology, University Hospital Zurich, Rämistrasse 100, CH-8091 Zürich, Switzerland; Department of Pulmonology, University Hospital Zurich, Rämistrasse 100, CH-8091 Zürich, Switzerland; Department of Pulmonology, University Hospital Zurich, Rämistrasse 100, CH-8091 Zürich, Switzerland; Department of Pulmonology, University Hospital Zurich, Rämistrasse 100, CH-8091 Zürich, Switzerland; Department of Pulmonology, University Hospital Zurich, Rämistrasse 100, CH-8091 Zürich, Switzerland; Department of Pulmonology, University Hospital Zurich, Rämistrasse 100, CH-8091 Zürich, Switzerland; Department of Pulmonology, University Hospital Zurich, Rämistrasse 100, CH-8091 Zürich, Switzerland

**Keywords:** Pulmonary hypertension, Pulmonary vascular disease, High altitude, Hypoxia, Oxygen

## Introduction

Stable-treated patients with pulmonary vascular disease (PVD) defined as pulmonary arterial or distal chronic thromboembolic pulmonary hypertension (PAH/CTEPH) wish to participate in daily activities including travel to high altitude or by air, but may be at increased risk of adverse events at high altitude (AE_HA_).^1,2^ Thus, pulmonary hypertension (PH) guidelines recommend that symptomatic PVD patients should not travel >1500 m or fly without supplemental oxygen therapy (SOT),^3^ but evidence is scarce and ambiguous.^4^

## Methods

Pulmonary vascular disease patients were investigated at 470 m (Zurich, low altitude, LA) and 2500 m (high altitude, HA) during a 30 h overnight stay at Mount Säntis according to a randomized-sequence, cross-over design (LA–HA vs. HA–LA) including >2-week washout between study sites. Patients were transported within 3 h by car and ropeway from home to 2500 m. The trial was performed between October 2021 and April 2022, ethically approved and registered (ClinicalTrials.gov: NCT05107700).

Patients were adults of all genders diagnosed with pre-capillary PH, classified as PAH or distal CTEPH,^3^ stable on medical therapy and providing written informed consent. Chronic thromboembolic pulmonary hypertension patients were inoperable or had persistent PH after interventional therapy. Patients with baseline PaO_2_ <8 kPa, FEV_1_ or FVC <70%, significant concomitant diseases, pregnancy or breast feeding were excluded.

The main outcomes were AE_HA_, pre-defined as (i) severe hypoxaemia [oxygen saturation by pulse oximetry (SpO_2_) < 80% > 30 min], or (ii) acute mountain sickness (AMS) by Lake Louise score ≥4 with headache or AMSc score ≥0.7, or (iii) any new illness including symptomatic cardiac arrhythmia, severe rise in systolic/diastolic blood pressure (>200/100 mmHg) or angina, and the therapeutic effect of SOT to restore baseline values in AE_HA_.

Participants’ SpO_2_ and condition were frequently monitored during daytime and continuously overnight at 2500 m. Patients with severe hypoxaemia were treated with SOT 3 L/min via nasal cannula (EverFlow, Philips Respironics) and had further HA assessments using SOT.

Other outcomes the second day at 2500 vs. 470 m were change in resting SpO_2_, systolic pulmonary artery pressure (sPAP) calculated from tricuspid regurgitation pressure velocity by echocardiography and 6 min walk distance (6MWD) including SpO_2_, heart rate, and Borg dyspnoea scale (Borg) at end-walk. Statistical analysis was performed with Stata (StataCorp, College Station, TX, USA) using mixed-linear regression models, and variables are presented as means ± standard deviation, numbers or mean differences (95% confidence intervals). A two-sided *P*-value <.05 was considered statistically significant.

## Results

**Figure 1:**
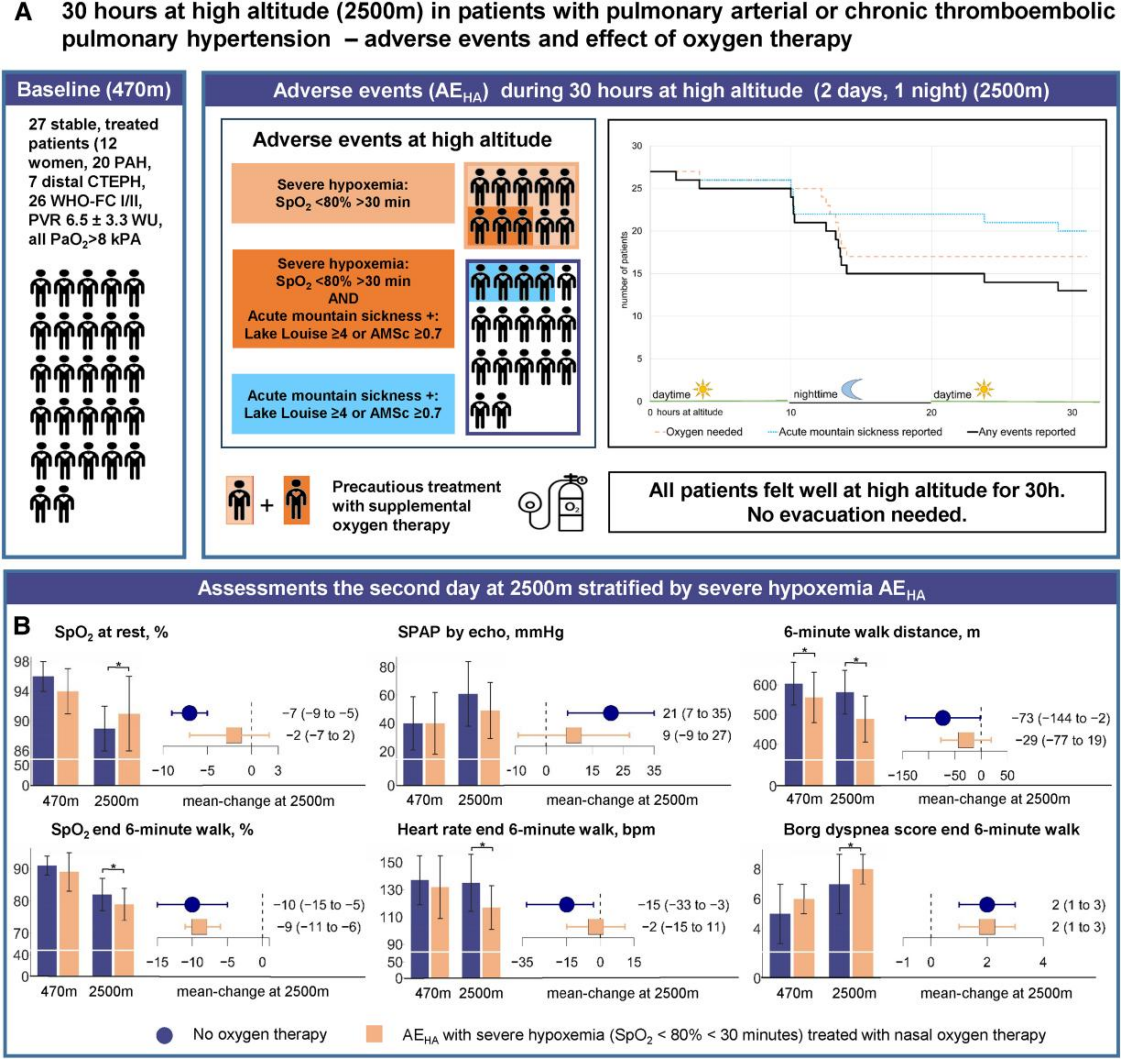
(*A*) Baseline characteristics of 27 patients with pulmonary vascular disease defined as pulmonary arterial hypertension or distal chronic thromboembolic pulmonary hypertension are shown (left sector, WHO-FC, WHO dyspnoea functional class; PVR, pulmonary vascular resistance; PaO_2_, partial pressure of oxygen in arterial blood). Adverse events at high altitude (AE_HA_) including severe hypoxaemia and acute mountain sickness are graphically illustrated (middle sector). The sector on the right reveals the time to AE_HA_ overall (solid black line) and stratified for patients with severe hypoxaemia treated with supplemental oxygen therapy (long dashed orange line) and those with acute mountain sickness without severe hypoxaemia (dotted blue line), with colours corresponding to the illustration in the middle sector. (*B*) Assessments at 470 and 2500 m are shown stratified for patients without supplemental oxygen therapy at 2500 m (left dark blue bars and whiskers) and patients with severe hypoxaemia AE_HA_ at 2500 m (right orange bars and whiskers). Bars at the left of each of the six subpanels represent means and standard deviations, whiskers represent mean difference and 95% confidence interval. *Significant difference (*P* < .05) as calculated by a mixed-linear regression model by intervention (2500 vs. 470 m) corrected for the intervention sequence as a fixed effect and subjects as a random effect. SPAP, systolic pulmonary artery pressure assessed from tricuspid regurgitation velocity without adding right atrial pressure by echocardiography

Of 65 assessed, 27 patients (12 women, 20 PAH, 7 distal CTEPH, age 62 ± 14 years) were included (*Figure 1*). All felt well during the study including 3 months thereafter. Pre-defined AE_HA_ occurred in 14/27: 10/27 (37%) had severe hypoxaemia (9/10 nocturnal), thereafter treated with SOT, and 7/27 scored positive for AMS, of which 3/7 combined with severe hypoxaemia.

In patients without severe hypoxaemia at 2500 vs. 470 m, the SpO_2_ was lower (89 ± 3 vs. 96 ± 2%, *P* < .001), the sPAP was higher (61 ± 23 vs. 40 ± 19 mmHg, *P* = .004), and 6MWD was equal (576 ± 74 vs. 605 ± 72 m, *P* = .240) with lower SpO_2_ (82 ± 5 vs. 91 ± 3%, *P* < .001), higher Borg (7 ± 2 vs. 7 ± 2, *P* < .001), but unchanged heart rate (135 ± 21 vs. 137 ± 18 b.p.m., *P* = .741) at end-walk. In patients with severe hypoxaemia needing SOT, SpO_2_ and sPAP were restored to baseline (91 ± 5 vs. 94 ± 3%, *P* = .250 and 49 ± 20 vs. 40 ± 22 mmHg, *P* = .322), whereas the 6MWD was reduced (485 ± 78 vs. 558 ± 85 m, *P* = .045) with a lower SpO_2_ (79 ± 5 vs. 89 ± 6%, *P* < .001), higher Borg (8 ± 1 vs. 6 ± 1, *P* < .001) but unchanged heart rate (117 ± 16 vs. 132 ± 23 b.p.m., *P* = .095).

## Discussion

This randomized controlled trial in stable PVD patients, mostly in functional Class I/II, showed that during 30 h at 2500 m in accordance with a weekend getaway, pre-defined AE_HA_ occurred in 14/27 patients, mostly as severe hypoxaemia during the night, which was effectively treated with subsequent SOT.^5^ All patients felt subjectively well at 2500 m. The threshold of SpO_2_ desaturation which forces physicians to provide SOT at HA is debated, but strongly depends on whether healthy or patients are concerned. Whilst anaesthetists maintain SpO_2_ >92% by routinely administering high-dose SOT, many tourists and mountaineers feel well for prolonged times at very HA with much lower SpO_2_ even under strenuous exercise.^6^ Healthy report SpO_2_ around 85% at 4500 m that decreases to 45% at 8848 m without experiencing long-term consequences. However, observing PVD patients with SpO_2_ <80% at HA would be considered hazardous by physicians and ethical board in fear of serious AE_HA_ and guidelines recommend to treat PVD patients with PaO_2_ <8 kPa with SOT.^3^ We thus administered SOT if SpO_2_ dropped <80% >30 min at HA, a threshold proven confident in >300 chronic obstructive pulmonary disease (COPD) patients, preliminary data in PVD and by the present PVD cohort.^1,5,7^

Data about PVD patients systematically investigated in a hypoxic environment are scarce and restricted to very short time, mostly to normobaric hypoxia.^1,2,8^ During a daytrip to 2500 m, 3/30 PVD patients needed SOT according to similarly defined AE_HA_.^1^ Presently, severe hypoxaemia occurred almost exclusively during nights, which is in accordance with COPD patients at 2590 m and a pilot of 9 PVD patients at 2048 m.^8^

Of the 7/27 PVD patients who scored positive for AMS due to mild headache, only 3 needed SOT, none wanted painkillers and all stayed at HA without subjective complaints. Thus, current AMS questionnaires may not define clinically relevant altitude illness in PVD patients, who may indicate occasional headache even at low altitude, potentially associated with vasodilator treatment. Albeit PVD patients with AE_HA_ were less fit, logistic regression models did not identify low-altitude measures that significantly predict AE_HA_.

At 2500 m, SOT restored SpO_2_ and sPAP to low-altitude levels, whereas patients not needing SOT revealed lower SpO_2_ and higher sPAP, but revealed similar 6MWD, suggesting unchanged pressure flow.^9^ Contrarily, patients needing SOT at 2500 m revealed a lower 6MWD along with a reduced SpO_2_ and higher Borg at end-walk. Thus, in contrast to high-flow SOT,^10^ 3 L/min via nasal cannula was not sufficient to reverse 6MWD and exercise-induced hypoxaemia at HA, potentially due to increased mouth breathing or insufficient flow. The main limitations are the relatively fit PVD collective with a mean baseline 6MWD of 580 m, the short HA exposure, and studying only central European PVD patients.

To conclude, stable, low-risk PVD patients tolerated a weekend getaway to 2500 m for up to 30 h generally well, none needed evacuation. In case of severe hypoxaemia, SOT restores resting but not exercise low-altitude physiology. The results of this field study help to counsel PVD patients for HA sojourns and call for future longer term studies.

## Data Availability

The data presented in this study are available on reasonable request from the corresponding author. Data will be uploaded to an online repository after publication including substudies.

## Pre-registered Clinical Trial Number

The pre-registered clinical trial numbers are (ClinicalTrials.gov: NCT05107700 and NCT05112172).
